# Supplementary material for: Transfer Hydrogenation of Vinyl Arenes and Aryl Acetylenes with Ammonia Borane Catalyzed by Schiff Base Cobalt(II) Complexes
Source: Int J Mol Sci. 2024 Apr 15;25(8):4363. doi: 10.3390/ijms25084363 (PMC11050580; doi:10.3390/ijms25084363)
Supplement: Supplementary file 1 [file ijms-25-04363-s001.zip › ijms-2932408-supplementary.pdf]

Supplementary Materials

for

Transfer Hydrogenation of Vinyl Arenes and Aryl Acetylenes with Ammonia Borane Catalyzed by Schiff Base Cobalt(II) Complexes

Maciej Skrodzki [1,2], Maciej Zaranek [2], Giuseppe Consiglio [3,*] and Piotr Pawluć [1,2,*]

[1] Faculty of Chemistry, Adam Mickiewicz University, Uniwersytetu Poznańskiego 8, 61-614 Poznań, Poland; maciej.skrodzki@amu.edu.pl
[2] Centre for Advanced Technologies, Adam Mickiewicz University, Uniwersytetu Poznańskiego 10, 61-614 Poznań, Poland; m.zaranek@amu.edu.pl
[3] Department of Chemical Science, University of Catania, Via S. Sofia 64, 95125 Catania, Italy
* Correspondence: giuseppe.consiglio@unict.it (G.C.); piotrpaw@amu.edu.pl (P.P.)

GCMS analyses of selected products

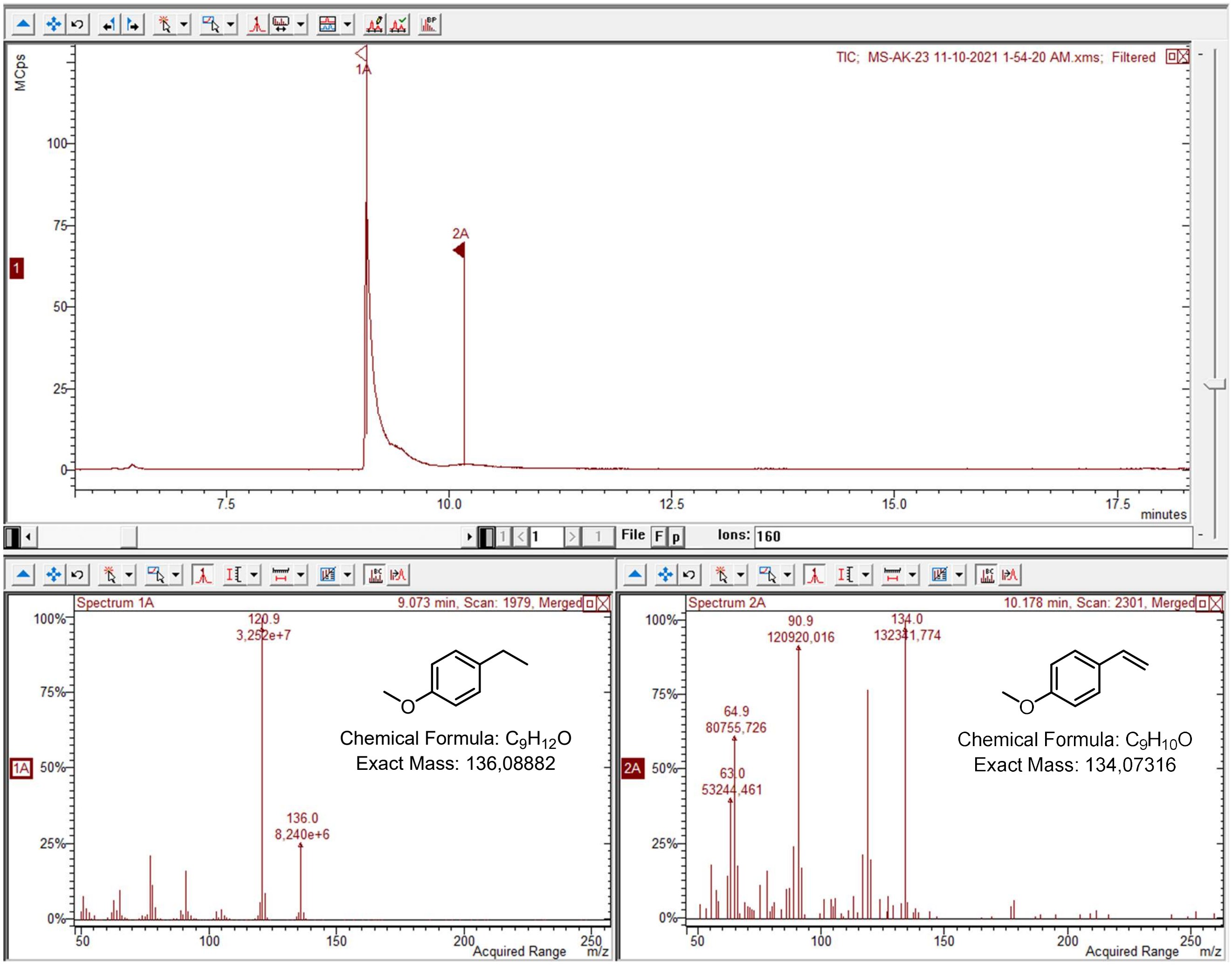

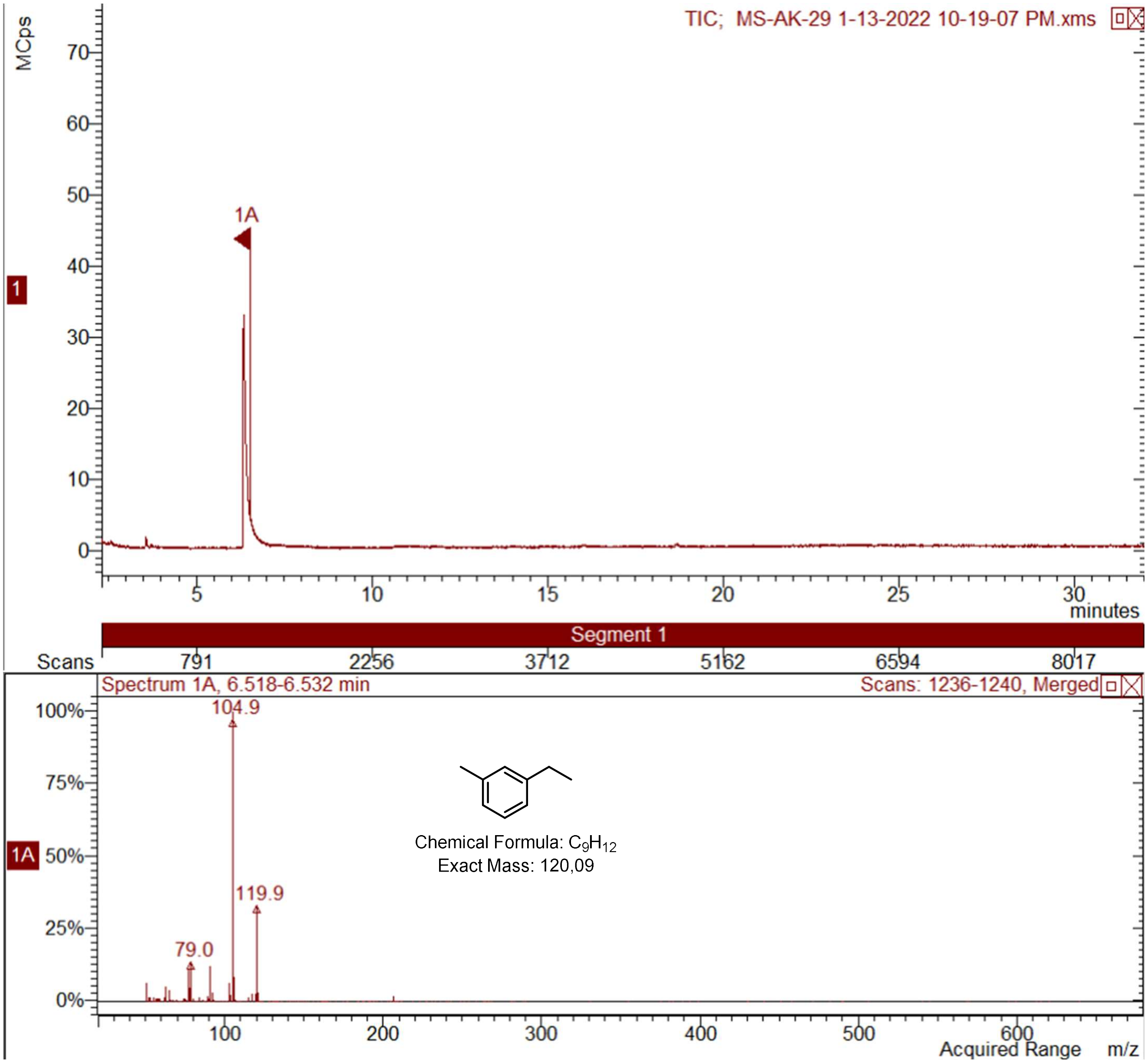

TIC; MS-AK-29 1-13-2022 10-19-07 PM.xms
MCps
70
60
50
40
30
20
10
0
1A
1
5
10
15
20
25
30
minutes
Segment 1
Scans
791
2256
3712
5162
6594
8017
Spectrum 1A, 6.518-6.532 min
Scans: 1236-1240, Merged
100%
75%
50%
25%
0%
1A
104.9
119.9
79.0
Chemical Formula: C_9H_{12}
Exact Mass: 120,09
100
200
300
400
500
600
Acquired Range
m/z

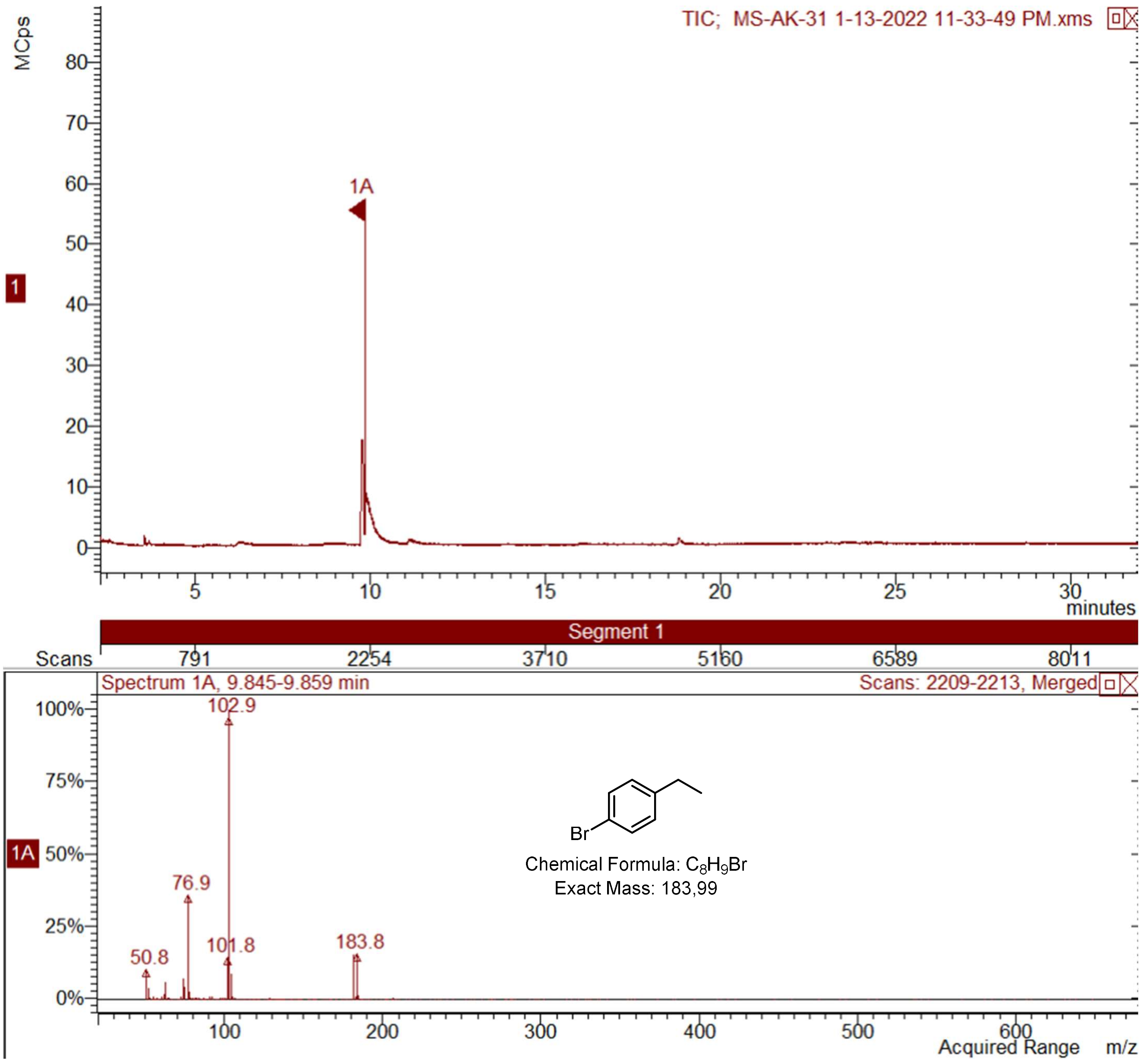

TIC; MS-AK-31 1-13-2022 11-33-49 PM.xms
MCps
1A
minutes
Segment 1
Scans
Spectrum 1A, 9.845-9.859 min
Scans: 2209-2213, Merged
102.9
76.9
50.8
101.8
183.8
Br
Chemical Formula: C8H9Br
Exact Mass: 183,99
Acquired Range
m/z

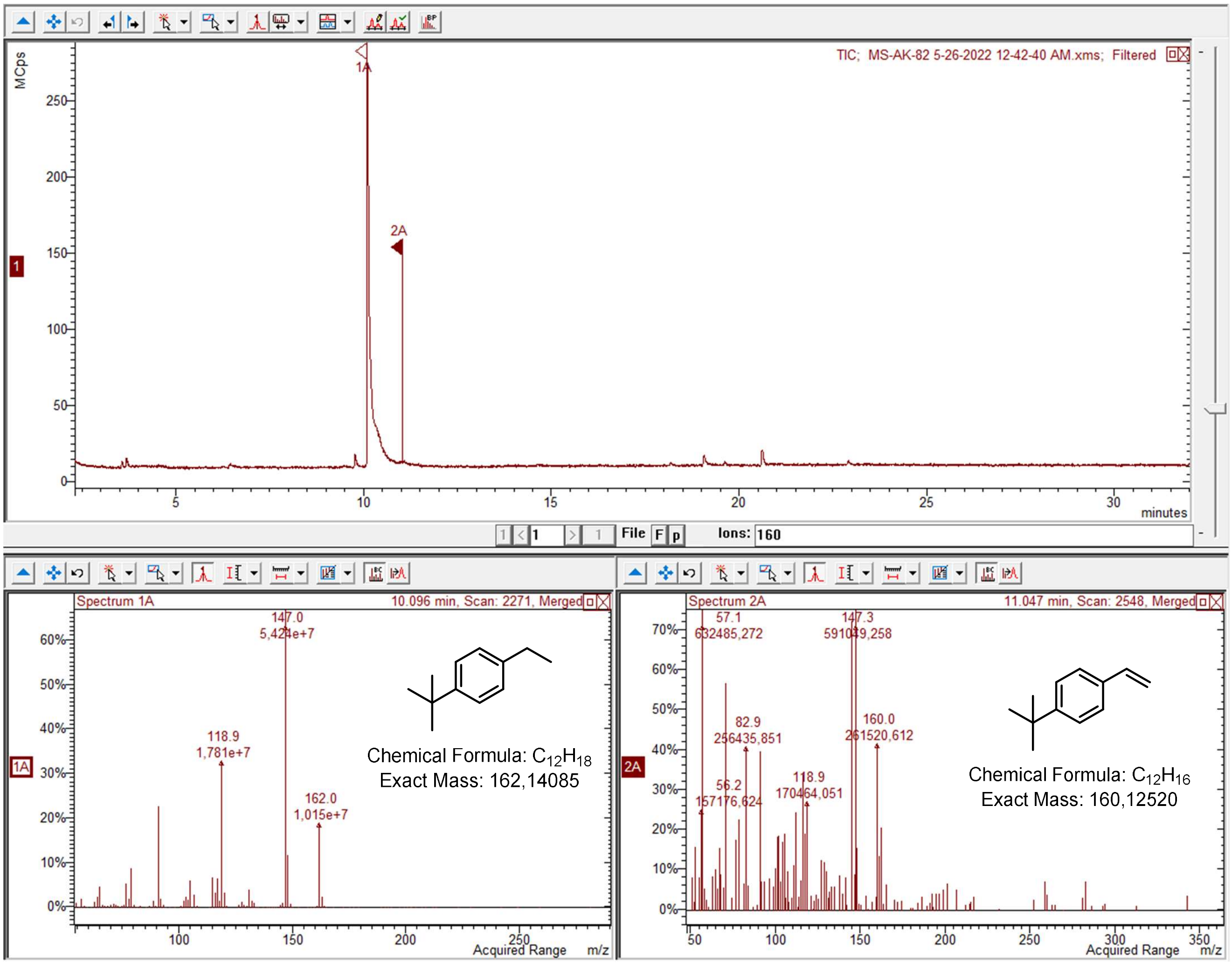
TIC; MS-AK-82 5-26-2022 12-42-40 AM.xms; Filtered
MCps
1A
2A
minutes
Ions: 160
Spectrum 1A
10.096 min, Scan: 2271, Merged
147.0
5,424e+7
118.9
1,781e+7
162.0
1,015e+7
Chemical Formula: C12H18
Exact Mass: 162,14085
Acquired Range
m/z
Spectrum 2A
11.047 min, Scan: 2548, Merged
57.1
632485,272
147.3
591039,258
82.9
256435,851
160.0
261520,612
56.2
157176,624
118.9
170464,051
Chemical Formula: C12H16
Exact Mass: 160,12520
Acquired Range
m/z

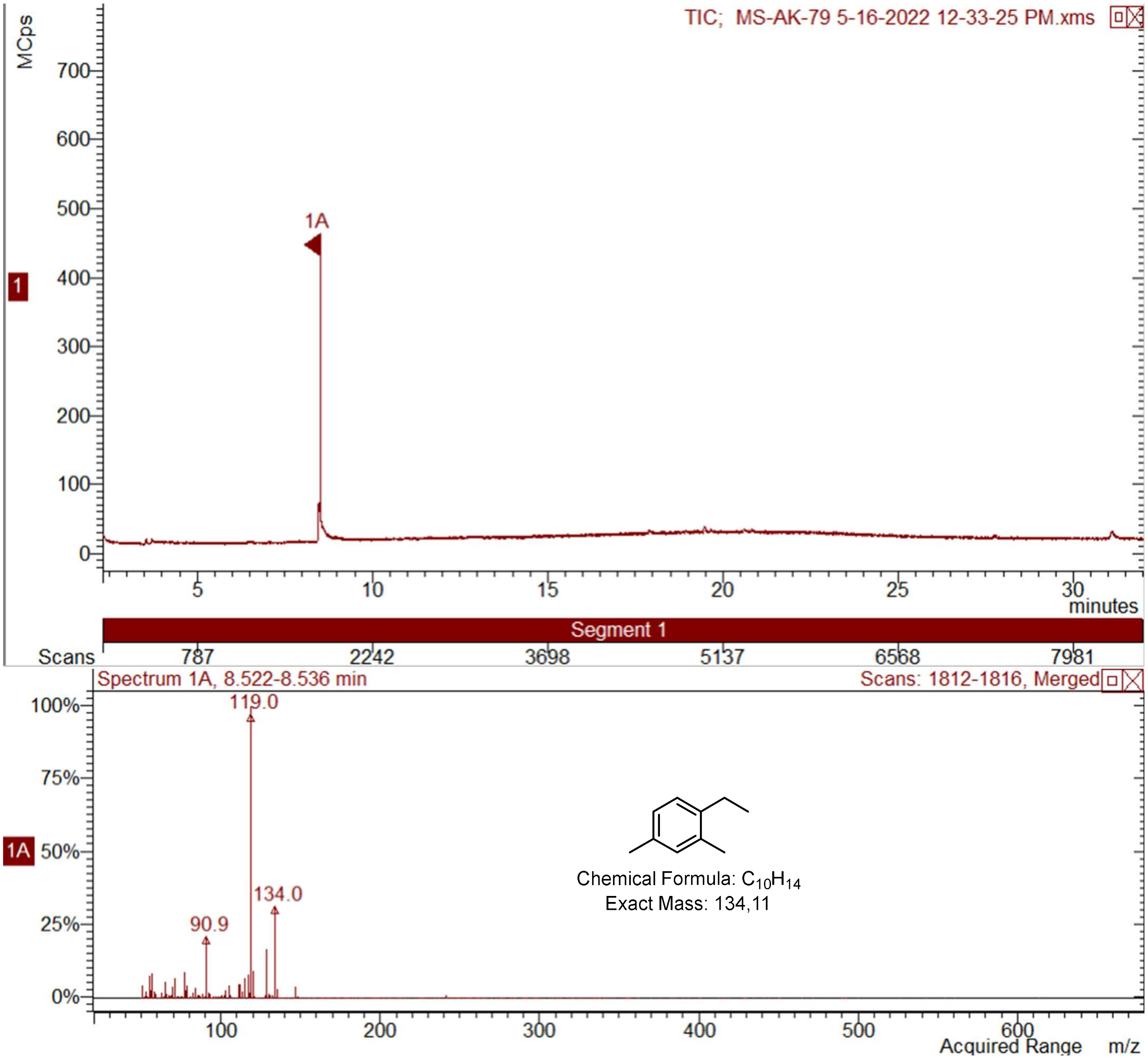

TIC; MS-AK-79 5-16-2022 12-33-25 PM.xms
MCps
1A
1
minutes
Segment 1
Scans
787
2242
3698
5137
6568
7981
Spectrum 1A, 8.522-8.536 min
Scans: 1812-1816, Merged
119.0
134.0
90.9
Chemical Formula: C10H14
Exact Mass: 134,11
Acquired Range
m/z

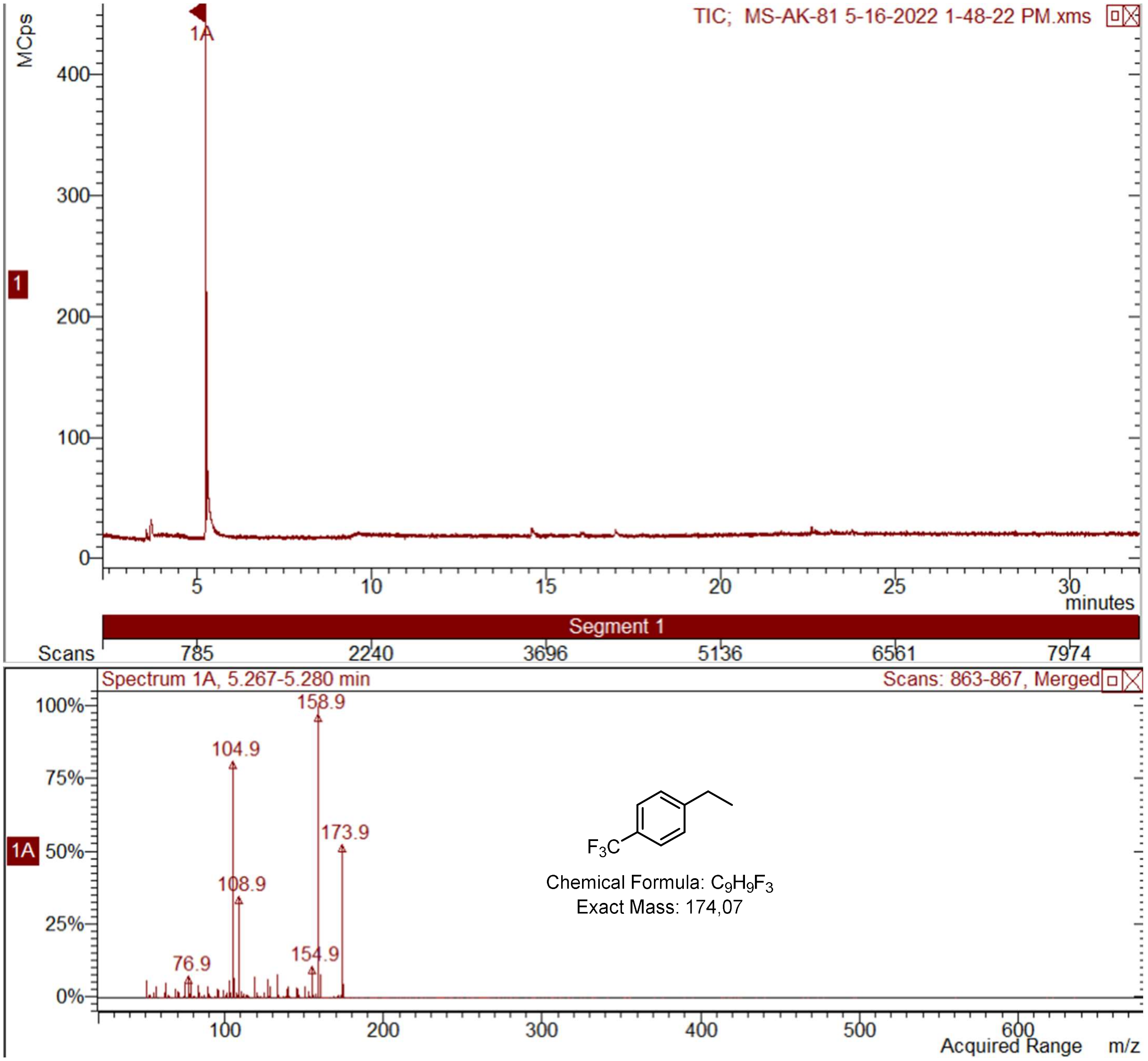
TIC; MS-AK-81 5-16-2022 1-48-22 PM.xms
MCps
400
300
200
100
0
1A
1
5
10
15
20
25
30
minutes
Segment 1
Scans
785
2240
3696
5136
6561
7974
Spectrum 1A, 5.267-5.280 min
Scans: 863-867, Merged
100%
75%
50%
25%
0%
1A
158.9
104.9
173.9
108.9
154.9
76.9
F3C
Chemical Formula: C9H9F3
Exact Mass: 174,07
100
200
300
400
500
600
Acquired Range
m/z

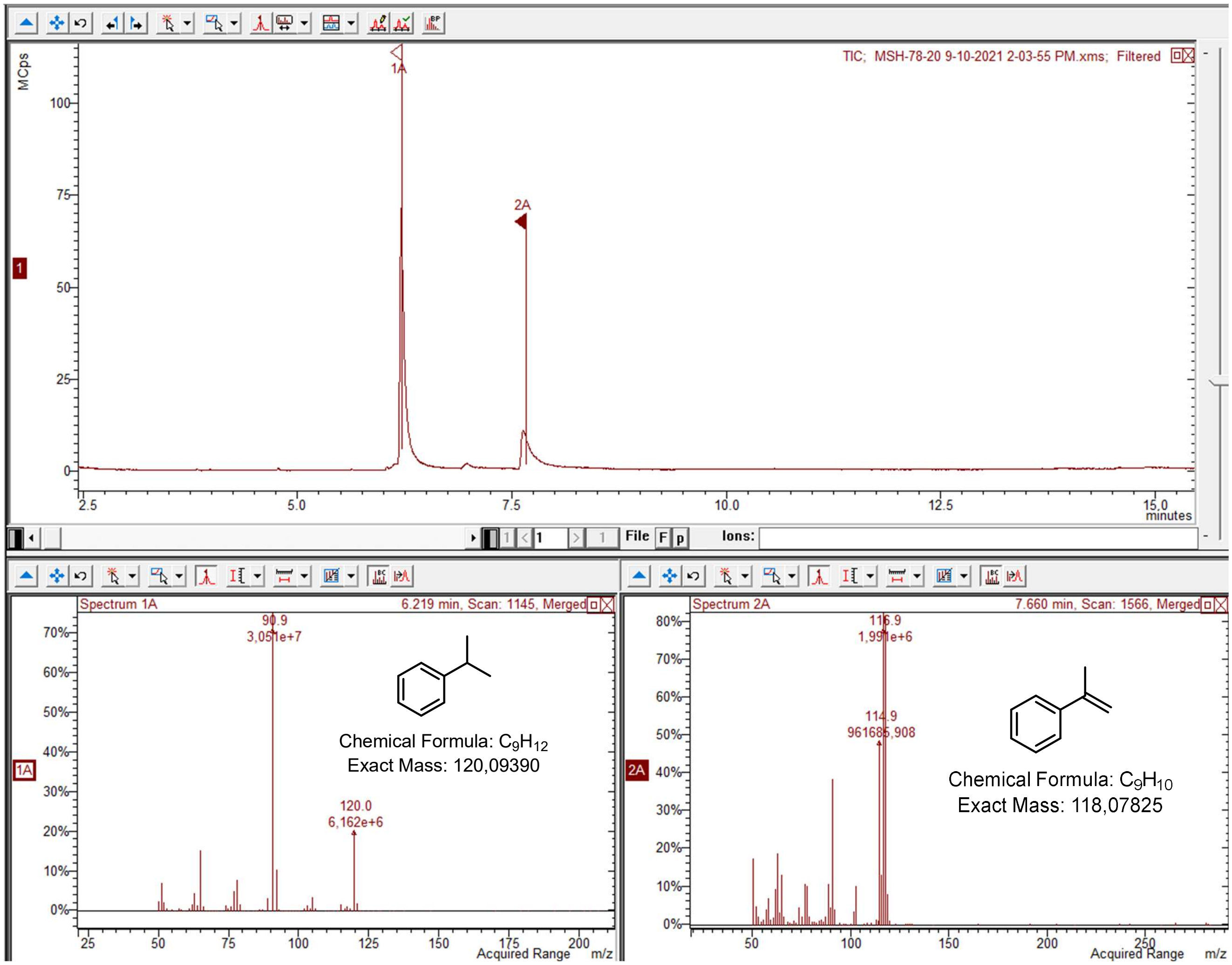
TIC; MSH-78-20 9-10-2021 2-03-55 PM.xms; Filtered
MCps
1A
2A
minutes
File F p
Ions:
Spectrum 1A
6.219 min, Scan: 1145, Merged
90.9
3,051e+7
120.0
6,162e+6
Chemical Formula: C_9H_{12}
Exact Mass: 120,09390
Acquired Range
m/z
Spectrum 2A
7.660 min, Scan: 1566, Merged
116.9
1,991e+6
114.9
961686,908
Chemical Formula: C_9H_{10}
Exact Mass: 118,07825
Acquired Range
m/z

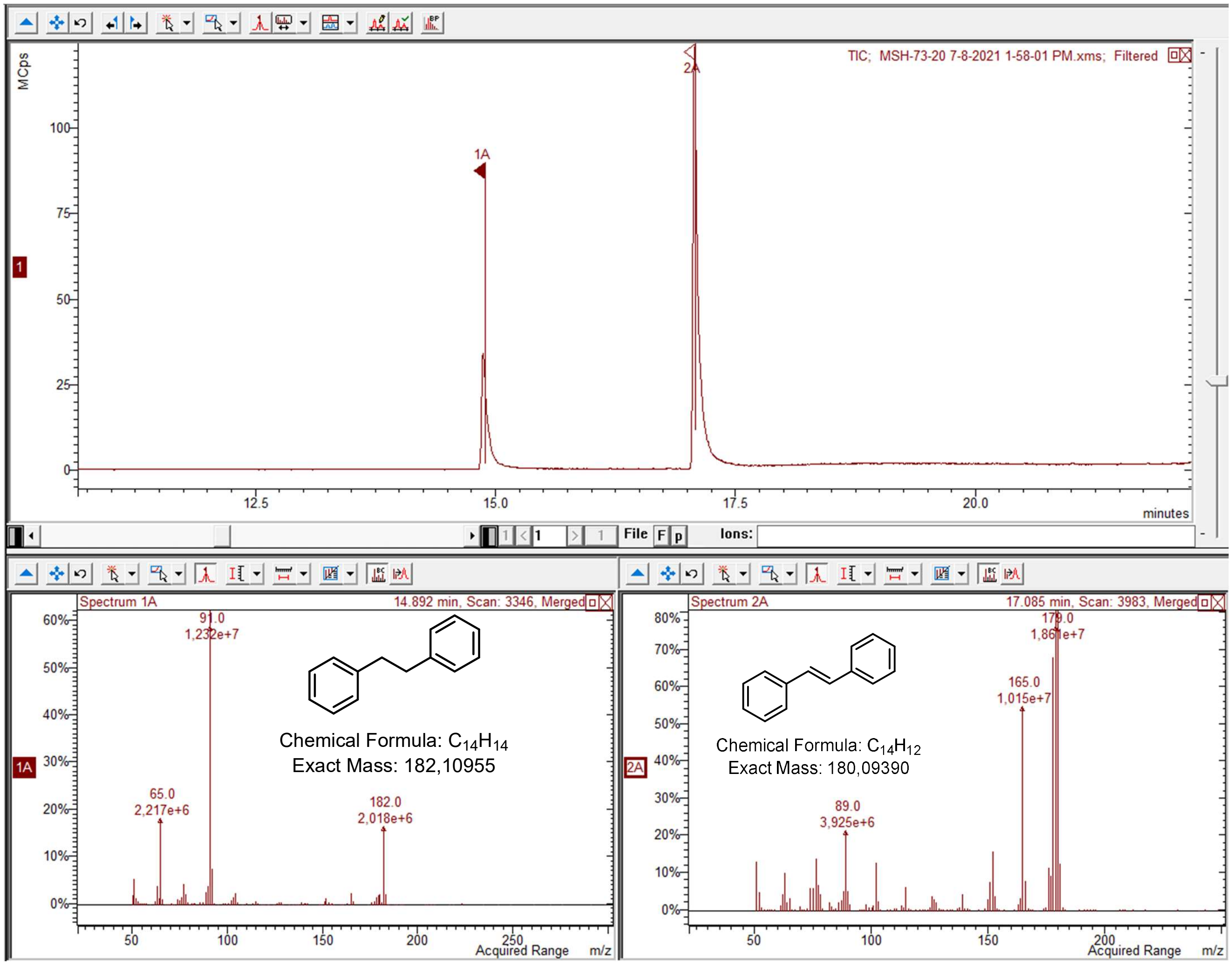

Setup and parameters of GC-MS: Bruker Scion 436-GC compiled with Bruker Scion SQ-MS. Analytical capillary column WCOT, diameter 0.25 mm, length 30 m, stationary phase Agilent DB-5. Method parameters: Injector temperature 240 ˚C, sample volume 1 µL, constant gas flow 1 mL/min, temperature program 60 ˚C (3 min); 10 ˚C/min; 250˚C (10 min).
